# Pregnancy-related low back pain and pelvic girdle pain approximately 14 months after pregnancy – pain status, self-rated health and family situation

**DOI:** 10.1186/1471-2393-14-48

**Published:** 2014-01-25

**Authors:** Cecilia Bergström, Margareta Persson, Ingrid Mogren

**Affiliations:** 1 Department of Clinical Sciences, Obstetrics and Gynecology, Umeå University, Umeå, Sweden; 2 School of Health and Social Studies, Dalarna University, Falun, Sweden

## Abstract

**Background:**

Pelvic girdle pain (PGP) in pregnancy is distinct from pregnancy-related low back pain (PLBP). However, women with combined PLBP and PGP report more serious consequences in terms of health and function. PGP has been estimated to affect about half of pregnant women, where 25% experience serious pain and 8% experience severe disability. To date there are relatively few studies regarding persistent PLBP/PGP postpartum of more than 3 months, thus the main objective was to identify the prevalence of persistent PLBP and PGP as well as the differences over time in regard to pain status, self-rated health (SRH) and family situation at 12 months postpartum.

**Methods:**

The study is a 12 month follow-up of a cohort of pregnant women developing PLBP and PGP during pregnancy, and who experienced persistent pain at 6 month follow-up after pregnancy. Women reporting PLBP/PGP (*n* = 639) during pregnancy were followed up with a second questionnaire at approximately six month after delivery. Women reporting recurrent or persistent LBP/PGP at the second questionnaire (*n* = 200) were sent a third questionnaire at 12 month postpartum.

**Results:**

A total of 176 women responded to the questionnaire. Thirty-four women (19.3%) reported remission of LBP/PGP, whereas 65.3% (*n* = 115) and 15.3% (*n* = 27), reported recurrent LBP/PGP or continuous LBP/PGP, respectively. The time between base line and the 12 months follow-up was in actuality 14 months. Women with previous LBP before pregnancy had an increased odds ratio (OR) of reporting ‘recurrent pain’ (OR = 2.47) or ‘continuous pain’ (OR = 3.35) postpartum compared to women who reported ‘no pain’ at the follow-up. Women with ‘continuous pain’ reported statistically significant higher level of pain at all measure points (0, 6 and 12 months postpartum). Non-responders were found to report a statistically significant less positive scoring regarding relationship satisfaction compared to responders.

**Conclusions:**

The results from this study demonstrate that persistent PLBP/PGP is a major individual and public health issue among women 14 months postpartum, negatively affecting their self-reported health. However, the perceived relationship satisfaction seems to be stable between the groups.

## Background

Historically, there have been discrepancies in regard to terminology regarding pelvic pain and/or low back pain in the pregnant population, but currently most adhere to the definition of pelvic girdle pain (PGP) suggested by Vleeming et al. [1]. Wu et al. [2] initially proposed the terms PGP and pregnancy-related low back pain (PLBP) where PGP is distinct from PLBP. PGP has been defined as “…pain is experienced in-between the posterior iliac crest and the gluteal fold, particularly in the vicinity of the sacroiliac joints (SIJ). The pain may radiate into the posterior thigh and can also occur in conjunction with/or separately in the symphysis pubis.” [1]. PLBP is instead characterized by a dull pain [3,4] and is more pronounced in forward flexion with associated restriction in spine movement and palpation of the erector spinae muscles exacerbates pain [5]. Hence, PLBP resembles LBP that occurs in a non-pregnant state.

PGP in pregnancy has been estimated to affect about half of pregnant women [2], where 25% experience serious pain and 8% experience severe disability [6]. This can be compared to 6.3% among non-pregnant women in the same age group [7]. Although PLBP and PGP are the most common complications of pregnancy, the underlying aetiology remains unknown. PLBP and PGP usually start around the 18th week of pregnancy, and usually reach its peak around week 24th and 36th of pregnancy. However, it may start as early as the first trimester or be delayed as late as 3 weeks after delivery [2,8]. Robinson et al. [9] identified five subgroups of self-rated pain locations in the pelvic area where women with combined symphysis pain and bilateral posterior pain are more afflicted compared to women with other pain combinations [9]. In addition, women with combined PLBP and PGP report more serious consequences in terms of health and function [8].

Even though most women who experience PLBP/PGP will recover postpartum, self-rated health (SRH) status appear to be less favourable and sexual life was less satisfactory in women with PLBP/PGP 6 months after pregnancy [10]. Furthermore, persistent pain and disability postpartum has been estimated to 7% [2]. Considering that recurrence of LBP in the general population is strongly correlated with previous episodes of LBP [11-15], PLBP/PGP may represent a specific risk factor for future persistent non-specific low back pain (NSLBP) [16].

To date there are relatively few studies regarding persistent PLBP/PGP postpartum of more than 3 months, and it would thus be of interest to gather more information regarding prevalence and disability in this particular group of women [6,16-19]. Hence, the primary aim of this study was to identify the prevalence of persistent PLBP and PGP at 12 months postpartum in regard to pain status, SRH and family situation. The secondary aim was to look at differences over time concerning pain status, SRH and family situation between women who reported ‘no-pain’ , ‘recurrent pain’ , and ‘continuous pain’ at the 12 months follow-up.

## Methods

The current study is a 12 month follow-up of a cohort of pregnant women developing PLBP and PGP during pregnancy [20], and who experienced persistent pain at 6 months follow-up after pregnancy [21]. In the primary population-based study with a cross-sectional design, all women who delivered from 1 January 2002 to 30 April 2002 at the Departments of Obstetrics and Gynaecology at Umeå University Hospital (UUH), county of Västerbotten, and Sunderby Hospital (SH), county of Norrbotten in northern Sweden, were invited to complete a questionnaire (questionnaire 1 = Q1) on their obstetric and gynaecological history, actual pregnancy and delivery [20]. The net sample consisted of 891 respondents (Q1) with a response rate of 83.2%. Detailed information on the sample has been presented in a previous publication [20].

**Figure 1:**
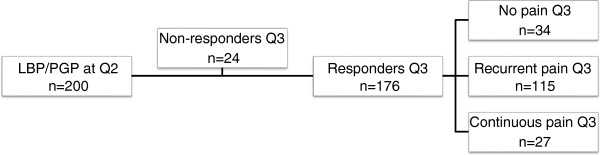
Selection chart of the study group.

Women reporting PLBP/PGP during pregnancy (Q1, *n* = 639) were followed up with a second questionnaire (Q2) at approximately six months after delivery, thus constituting a cohort. Only women reporting PLBP/PGP during pregnancy were followed up, as PLBP/PGP only arises during pregnancy. Further, the current study only investigated factors related to the remission or persistency of PLBP/PGP postpartum. The Q2 included issues such as remission or persistency of PLBP/PGP 6 months after pregnancy, use of medical services, family situation, SRH, sick leave, sex life, physical activities, oral contraception and breastfeeding. The net sample comprised 464 women who responded to the Q2 constituting a response rate of 72.6%. Further details of this sample have been previously reported in several papers [10,21,22]. At the six month follow-up (Q2), 43.1% (*n* = 200) still experienced recurrent or persistent PLBP/PGP after pregnancy. These women (*n* = 200) were followed-up with a third questionnaire (Q3) at approximately 12 months postpartum. Q3 included many of the similar issues as in Q2. Q3 was sent to all eligible women within an interval of 2 weeks before or 2 weeks after 12 months postpartum. At least one reminder was sent to all eligible subjects. A total of 176 women, out of 200, responded to Q3, thus giving a response rate of 88% which constitutes the final sample of this study. The final sample for this study is presented in Figure 1.

### Definitions

*PLBP or PGP* in Q1 was defined as ‘recurrent pain’ or ‘continuous pain’ for more than 1 week of the lumbar spine or pelvis during the recent pregnancy. A woman was considered to be suffering from PLBP/PGP during pregnancy if she responded positively to the specific question regarding localisation of pain, which also included marking the affected area on a drawing included in the questionnaire [20].

*Actual PLBP/PGP* postpartum was defined as a positive response if the women reported *actual* PLBP and/or PGP in Q2 and Q3. The response alternatives to the question ‘Do you experience low back pain or pelvic pain right now’? were ‘yes, recurrent pain’ , ‘yes, continuous pain’ , and ‘no pain’. Women reporting a specific time point at which PLBP/PGP had ceased, even though reporting ‘recurrent pain’ , were allocated to the ‘no pain’ group [21,23,24].

*Persistent PLBP and PGP* after pregnancy included women who reported both ‘recurrent pain’ and ‘continuous pain’ defined and PLBP and/or PGP after pregnancy.

*Parity*. Number of births.

*Physical activity* in all three questionnaires (Q1-Q3) was reported through three questions and included: 1) Do you exercise/do sport on a regular basis right now? 2) If you exercise/do sports on a regular basis right now, how many times a week, on average, do you exercise/do sports? 3) If you exercise/do sport on a regular basis right now, how long after the pregnancy did you commence with regular exercise/sports? The response alternatives to question no. 1 was ‘yes’ or ‘no’ and the response alternative for question no. 2 was to fill in the average number of days and the response alternative for question no. 3 was to fill in the number of months postpartum when the subject started exercising. In addition, Q1 also included the question ‘What kind of sports/activities do you mainly take part in?’ where the respondents could respond with free text.

*Pain* was reported through a visual analogue scale (VAS) of 10 centimetres, where 0 denoted ‘no pain’ and 10 denoted ‘worst imaginable pain’. Women reporting a score ≥7.0 on the VAS 0-10 cm were considered to have severe pain [25,26]. This cut-off score has previously been used for the same study group [10,20]. The character of pain was assessed at Q2 and Q3. The options were ‘dull pain’ , ‘cutting pain’ , ‘burning pain’ , ‘stabbing pain’ , or ‘other pain’. It was possible to give more than one option. The change of character and its localisation was assessed at Q3 with the options ‘yes’ , ‘no’ , and ‘do not know’ on both questions.

*Self-rated health (SRH)*. The woman was asked to assess her health status before, during, and after pregnancy at all data collection periods (Q1, Q2, and Q3). A five category alternative was used in order to differentiate the response among the individuals and the options were: ‘very good’ , ‘quite good’ , ‘fair’ , ‘quite poor’ , and ‘poor’.

*Family situation.* The participants were asked about their marital status where the options were ‘single’ , ‘cohabiting’ , ‘relationship but not cohabiting’ , and ‘married’. Further, the woman was asked to grade their relationship at Q1, Q2 and Q3. The options where ‘very good’ , ‘good’ , ‘neither good nor bad’ , ‘bad’ , and ‘very bad’.

### Statistics

Analysis of the sample (Q3, *n* = 176) was done through calculation of means and standard deviations (SD) for parametric data. Independent-samples *t* test was used to test difference between groups for parametric data that also included analysis of difference between respondents and non-respondents when possible. To test for the difference between groups regarding non-parametric data, a non-parametric two-independent samples testing was used. Pearson χ^2^ test was used to test the difference between groups of categorical data, including analysis between respondents and non-respondents when pertinent.

Due to violation of normality assumption of the dependent variables (pain, SRH and relationship satisfaction), the non-parametric test Kruskal-Wallis one-way analysis-of-variance-by-ranks on ranks was used in order to test for the difference between three or more groups [27]. The Mann-Whitney *U* test was also applied to compare differences between two independent groups. The ‘no pain’ group was used as a predefined reference group in all analyses. Statistical significance was set at *p* < 0.05 when comparing differences between the groups. IBM SPSS Statistics 19 software package was used.

### Ethical approval and informed consent

The study was approved by the Ethics Committee at the Umeå University (Dnr 01-335). A written consent was obtained from all participants.

## Results

In total, 176 out of 200 eligible women responded to Q3, thus constituting the study group (Figure 1). A total of 34 women (19.3%) reported remission of PLBP/PGP at Q3, whereas a proportion of 65.3% (*n* = 115) and 15.3% (*n* = 27), reported recurrent PLBP/PGP or continuous LBP/PGP respectively. Fifty percent (*n* = 14) of women with ‘continuous pain’ at Q2 reported ‘recurrent pain’ at Q3 and 68% (*n* = 101) of women with ‘recurrent pain’ at Q2 still reported ‘recurrent pain’ at Q3. Close to 22% (*n* = 32) of women with ‘recurrent pain’ at Q2 reported ‘no pain’ at Q3 and about 7% (*n* = 2) of women reporting ‘continuous pain’ at Q2, reported ‘no pain’ at Q3. Nearly 43% (*n* = 12) of women with ‘continuous pain’ and 10% (*n* = 15) of women with ‘recurrent pain’ reported ‘continuous pain’ at Q3, respectively. Table 1 describes the study group at Q3, where the mean maternal age at Q1 was 30.7 years for all respondents, and mean age at filling in Q3 was 31.9 years, yielding a time distance of 14 months between Q1 and Q3. There were no statistically significant differences between the ‘no pain’ , ‘recurrent pain’ , and ‘continuous pain’ groups as well as between the respondents and non-respondents at Q3 for most variables in Table 1. However, a statistically significant difference was found between the ‘recurrent pain’ and the ‘continuous pain’ group in regard to smoking, where the ‘continuous pain’ group included significant more smokers compared to the ‘recurrent pain’ group (*p* = 0.03). Also, there was a significant difference in the reported maternal age at first delivery, with a higher reported age in the non-respondents compared to the respondents (mean 28.6 (SD 3.66), *p* = 0.017).

**Table 1:** Descriptive information of the study group at Q3

	**No pain**	**Recurrent pain**	**Continuous pain**	**Total**
	***n*** **=34**	***n*** **=115**	***n*** **=27**	***n*** **=176**
**Age,** mean (SD)	31.56 (4.9)	32.2 (4.6)	31.3 (5.7)	31.9 (4.8)
**Age at first delivery**, mean (SD)	26.2 (3.7)	25.8 (4.3)	24.7 (3.7)	25.7 (4.1)
**Marital status**				
Single	-	3 (2.6)	2 (7.4)	5 (2.8)
Cohabiting	24 (70.6)	71 (61.7)	12 (44.4)	107 (60.8)
Relationship but not cohabiting	-	1 (0.9)	1 (3.7)	2 (1.1)
Married	10 (29.4)	40 (34.8)	12 (44.4)	62 (35.2)
**Education at Q1**				
Compulsory school	2 (5.9)	3 (2.6)	1 (3.7)	6 (3.4)
High school	13 (38.2)	53 (46.1)	16 (59.3)	82 (46.6)
Folk high school	-	2 (1.7)	-	2 (1.1)
University	19 (55.9)	57 (49.6)	10 (37.0)	86 (48.9)
**Parity**				
1	16 (91.2)	37 (32.2)	12 (44.4)	65 (36.9)
2	9 (26.5)	48 (41.7)	10 (37.0)	67 (38.1)
3	8 (23.5)	24 (20.9)	3 (11.1)	35 (19.9)
≥4	1 (2.9)	6 (5.2)	2 (7.4)	9 (5.1)
**Physical activity**				
Yes	19 (55.9)	69 (60)	19 (70.4)	107 (60.8)
No	15 (44.1)	46 (40)	8 (29.6)	69 (39.2)
**Self-rated health (SRH)**				
Very good	7 (20.6)	18 (15.7)	2 (7.4)	27 (15.4)
Quite good	18 (52.9)	56 (48.7)	10 (37.0)	84 (48.0)
Fair	6 (17.6)	34 (29.6)	10 (37.0)	50 (28.6)
Quite poor	3 (8.8)	5 (4.3)	4 (14.8)	12 (6.9)
Poor	-	1 (0.9)	1 (3.7)	2 (1.1)
**Smoking/tobacco**				
Yes	3 (8.8)	7 (6.1)	6 (22.2)	16 (9.1)
No	31 (91.2)	107 (93.0)	21 (77.8)	159 (90.3)
Snuff	-	1 (0.9)	-	1 (0.6)

Numbers in parenthesis are percentage unless otherwise specified.

^1^‘No pain’ denotes respondents reporting remission of LBP/PGP at Q3.

^2^‘Recurrent pain’ denotes respondents reporting recurrent LBP/PGP at approximately 14 months post-partum at Q3.

^3^‘Continuous pain’ denotes respondents reporting continuous LBP/PGP at approximately 14 months post-partum at Q3.

### Pain status

**Figure 2:**
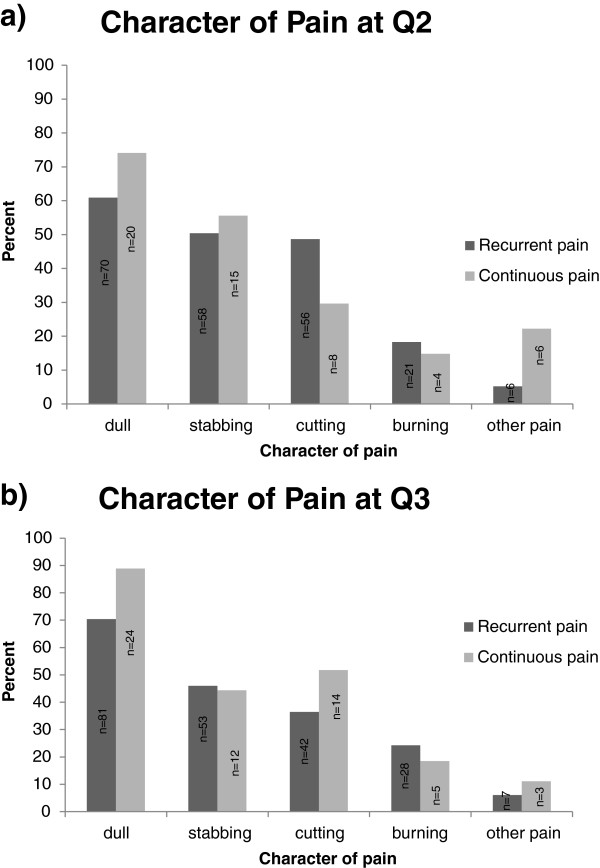
Character of pain at Q2 (a) and Q3 (b).

The majority (60%) of the women reported a dull pain at both Q2 and Q3. The second and third most common pain character reported at Q3 was ‘stabbing pain’ (46%) and ‘cutting pain’ (39%). Figure 2a and b gives an overview of character of pain at both Q2 and Q3 for both the ‘recurrent pain’ and ‘continuous pain’ group. Analysis showed that there was a statistically significant difference in pain character (dull, cutting, burning, and stabbing pain) at Q2 between the ‘recurrent pain’ and the ‘continuous pain’ group (*p* = 0.023 and *p* = 0.023 respectively), where the ‘recurrent pain’ group reported less dull and stabbing but reported more of a cutting and burning pain character. A statistically significant difference was also shown at Q2 between ‘recurrent pain’ and the ‘continuous pain’ group in regard to ‘other pain’ (*p* = 0.006). At Q3, a statistically significant difference in pain character was demonstrated between ‘recurrent pain’ and ‘continuous pain’ pain group pertaining to dull, cutting, burning, and stabbing pain (*p* = 0.021). Furthermore, a statistically significant difference was shown between the non-respondents and the respondents at Q2 regarding the character of pain concerning dull, cutting, burning, and stabbing pain (*p* = 0.003) as well as to ‘other pain’ (*p* < 0.001).

Close to 89% of the women in the ‘continuous pain’ group and 66% of the women in the ‘recurrent pain’ group reported no change in the localisation of pain (as reported in Q1) at Q3 compared to the first 6 months postpartum. However, there was a statistically significant difference between the ‘recurrent pain’ and ‘continuous pain group regarding change of the localisation of pain (as reported in Q1) at Q3, where almost 90% of women reporting ‘continuous’ pain reported no change of pain localisation compared to 66.1% of women with ‘recurrent’ pain (*p* = 0.036).

Table 2 gives an overview of previous back pain (before pregnancy), and pain sites in the three different groups (‘no pain’ , ‘recurrent pain’ and ‘continuous pain’) at Q3. The most common pattern of pain was mixed pain sites (73.3%), and the majority of women with ‘recurrent pain’ and ‘continuous pain’ had experienced previous LBP, while the majority of women in the ‘no pain’ group did not have any experiences with previous LBP. In regard to previous LBP, there was a statistically significant difference between women with ‘no pain’ and ‘recurrent pain’ χ2 (*n* = 147) = 4.78, *p* = 0.030, as well as between ‘no pain’ and ‘continuous pain’ χ2 (*n* = 60) = 5.07, *p* = 0.024 at Q3. The odds ratio (OR) of having ‘recurrent pain’ and ‘continuous pain’ in women who had experienced previous LBP compared to women who had not reported previous LBP was (with 95% confidence interval (CI) in parenthesis): OR = 2.47, (1.08 – 5.65), *p* = 0.033 and OR = 3.35, (1.15 – 9.73), *p* = 0.027 respectively.

**Table 2:** Descriptive information of the study group at Q3 regarding previous LBP, and pain site localisation as reported at Q1

	**No pain**^**1**^	**Recurrent pain**^**2**^	**Continuous pain**^**3**^	***p*-value**		
	**n= 34**	**n= 115**	**n= 27**	**1 vs. 2**	**1 vs. 3**	**2 vs. 3**
**Previous LBP** (before pregnancy)				0.030*	0.024*	0.482
Yes	10 (30.3)	59 (51.8)	16 (59.3)			
No	23 (69.7)	55 (48.2)	11 (40.7)			
**Pain sites reported at Q1,***n* (%)				0.112	0.256	0.984
Back	10 (29.4)	21 (18.3)	5 (18.5)			
Front	4 (11.8)	6 (5.2)	1 (3.7)			
Mixed (back and front)	20 (58.8)	88 (76.5)	21 (77.8)			

Test for difference between groups (Pearson’s chi-square).

^1^‘No pain’ denotes respondents reporting remission of LBP/PGP at Q3.

^2^‘Recurrent pain’ denotes respondents reporting recurrent LBP/PGP at approximately 14 months after pregnancy at Q3.

^3^‘Continuous pain’ denotes respondents reporting continuous LBP/PGP at approximately 14 months after pregnancy at Q3.

*Significance test *p*<0.05.

A Kruskal-Wallis one-way ANOVA rank test was performed on the results of the different groups (Table 3). The analysis showed a statistically significant effect on highest level of pain: a) during pregnancy Q1 (*p* = 0.007), b) the past 6 months postpartum Q2 (*p* < 0.001), c) pain the past week Q2 (*p* < 0.001), d) the past 6 months Q3 (*p* < 0.001), and e) pain the past week Q3 (*p* < 0.001) for both the ‘recurrent pain’ and ‘continuous pain’ group. When performing Mann-Whitney *U* test between groups, this significant statistical difference between the ‘no pain’ and the ‘continuous pain’ was still present. However, there was only a statistically significant difference between the ‘no pain’ and ‘recurrent pain’ group regarding: a) the past 6 months Q3 (5.3 (IR = 4.0), *p* < 0.001), and b) pain the past week Q3 (3.55 (IR = 4.1), *p* < 0.001).

**Table 3:** Comparing highest level of pain at Q1, Q2 and Q3 using women with ‘no pain’ at Q3 as the reference group

	**Recurrent pain Q3**				**Continuous pain Q3**				
**Highest level of pain**	**n**	**Median**^**a**^	**IR**^**b**^	***p*-value MW-test**	**n**	**Median**^**a**^	**IR**^**b**^	***p*-value MW-test**	***p*-value**^**c **^**KW-test**
Q1: During pregnancy	111	6.3	2.5	0.711	27	7.8	2.9	0.008*	0.007**
Q2: During the past 6 months	114	6.0	3.5	0.058	27	8.3	2.7	<0.001*	<0.001**
Q2: Past week	114	4.1	4.1	0.333	27	7.1	4.8	<0.001*	<0.001**
Q3: During the past 6 months	114	5.3	4.0	<0.001*	27	7.5	2.6	<0.001*	<0.001**
Q3: Past week	114	3.6	4.1	0.027*	27	6.6	2.9	0.005*	<0.001**

^a^Median Mann–Whitney U test. ^b^IR = Interquartile Range. ^c^Kruskal-Wallis *p*-value. *Significant result *p*<0.05 using Mann–Whitney *U* test.

**Significant result *p*<0.05 using Kruskal-Wallis test.

### Self-rated health (SRH)

**Figure 3:**
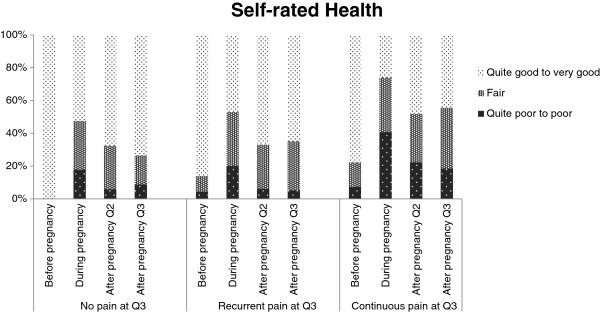
Self-rated health before and during pregnancy and at Q2 and Q3.

Figure 3 illustrates the patterns of SRH before pregnancy (Q1), during pregnancy (Q1), 6 months postpartum (Q2), and 14 months postpartum (Q3) for the three different subgroups, where the women in the ‘continuous pain’ group seem to report a less favourable health status (with the largest difference in SRH during pregnancy) compared to both the ‘recurrent pain’ and the ‘no pain’ group. Table 4 shows that there is a statistically significant difference between the ‘recurrent pain’ and the ‘continuous pain’ group (categorized at Q3) regarding SRH status during pregnancy (Q1) (*p* = 0.041) and during the first 6 months postpartum (Q2) (*p* = 0.050). Furthermore, the odds of women with ‘continuous pain’ at Q3 assessing their SRH status as poor or quite poor compared to women with ‘recurrent pain’ was (with 95% CI in parenthesis): OR = 1.54, (1.01 – 2.34), *p* = 0.043.

**Table 4:** Self-rated health

**Self-reported health (SRH)**	**All subjects**	**No pain**^**1**^	**Recurrent pain (LBP/PGP)**^**2**^	**Continuous pain (LBP/PGP)**^**3**^	**P-value**^**4**^			**Non-respondents**	
					**1 vs. 2**	**1 vs. 3**	**2 vs. 3**		**p-value**^**5**^
**Number of subjects** (%)	176 (100.0)	34 (19.3)	115 (65.3)	27 (15.3)				24	
**Before pregnancy Q1** n (%)									
Very good	62 (35.2)	10 (29.4)	43 (37.4)	9 (33.3)	0.056	0.052	0.284	9 (39.1)	0.820
Quite good	92 (52.3)	24 (70.6)	56 (48.7)	12 (44.4)				11 (47.8)	
Fair	15 (8.5)	-	11 (9.6)	4 (14.8)				3 (13.0)	
Quite poor	6 (3.4)	-	5 (4.3)	1 (3.7)				-	
Poor	1 (0.6)	-	-	1 (3.7)				-	
**During pregnancy Q1** n (%)									
Very good	25 (14.2)	6 (17.6)	17 (14.8)	2 (7.4)	0.961	0.099	0.041*	3 (13.0)	0.973
Quite good	54 (30.7)	12 (35.3)	37 (32.2)	5 (18.5)				8 (34.8)	
Fair	57 (32.4)	10 (29.4)	38 (33.0)	9 (33.3)				8 (34.8)	
Quite poor	27 (15.3)	5 (14.7)	17 (14.8)	5 (18.5)				3 (13.0)	
Poor	13 (7.4)	1 (2.9)	6 (5.2)	6 (22.2)				1 (4.3)	
**At 6 months post-partum Q2** n (%)									
Very good	31 (17.6)	7 (20.6)	22 (19.1)	2 (7.4)	0.726	0.223	0.050*	6 (25.0)	0.690
Quite good	82 (46.6)	16 (47.1)	55 (47.8)	11 (40.7)				9 (37.5)	
Fair	48 (27.3)	9 (26.5)	31 (27.0)	8 (29.6)				7 (29.2)	
Quite poor	9 (5.1)	2 (5.9)	3 (2.6)	4 (14.8)				2 (8.3)	
Poor	6 (3.4)	-	4 (3.5)	2 (7.4)				-	
**At 12 months post-partum Q3** n (%)									
Very good	27 (15.4)	7 (20.6)	18 (15.8)	2 (7.4)	0.538	0.166	0.132		
Quite good	84 (48.0)	18 (52.9)	56 (49.1)	10 (37.0)					
Fair	50 (28.6)	6 (17.6)	34 (29.8)	10 (37.0)					
Quite poor	12 (6.9)	3 (8.8)	5 (4.4)	4 (14.8)					
Poor	2 (1.1)	**-**	1 (0.9)	1 (3.7)					

Test for difference between groups at Q3 (Pearson’s chi-square).

^1^‘No pain’ denotes respondents reporting remission of LBP/PGP at Q3.

^2^‘Recurrent pain’ denotes respondents reporting recurrent LBP/PGP at approximately 14 months after pregnancy at Q3.

^3^‘Continuous pain’ denotes respondents reporting continuous LBP/PGP at approximately 14 months after pregnancy at Q3.

^4^‘No pain’ vs. ‘Recurrent pain’, ‘No pain’ vs. ‘Continuous pain’ and Recurrent pain’ vs. ‘Continuous pain’.

^5^Non-respondents vs. respondents.

*Significance test *p*<0.05.

### Relationship satisfaction

Evaluation of the individuals’ assessment (categories given at Q3) of their relationship before pregnancy (Q1), 6 months postpartum (Q2), and 12 months postpartum (Q3) is presented in Table 5. The results show a stable relationship satisfaction between the three subgroups, where the majority of individuals rated their relationship with their partner as ‘good’ or ‘very good’. However, there was a statistically significant difference in relationship satisfaction between non-respondents and respondents at Q1 (*p* = 0.021), where non-respondents reported poorer relationship satisfaction compared to the responders.

**Table 5:** Relationship satisfaction

**Relationship satisfaction**	**All subjects**	**No pain**^**1**^	**Recurrent pain (LBP/PGP)**^**2**^	**Continuous pain (LBP/PGP)**^**3**^	**P-value**^**4**^			**Non-respondents**	
					**1 vs. 2**	**1 vs. 3**	**2 vs. 3**		**p-value**^**5**^
**Number of subjects** (%)	176 (100.0)	34 (19.3)	115 (65.3)	27 (15.3)				24	
**Before pregnancy Q1**									
Very good	137 (78.3)	28 (82.4)	88 (77.2)	21 (77.8)	0.784	0.905	0.860	12 (52.2)	0.021*
Good	29 (16.6)	5 (14.7)	19 (16.7)	5 (18.5)				10 (43.5)	
Neither good or bad	6 (3.4)	1 (2.9)	4 (3.5)	1 (3.7)				1 (4.3)	
Bad	-	-	-	-				-	
Very bad	3 (1.7)	-	3 (2.6)	-				-	
**At 6 month post-partum Q2**									
Very good	84 (48.3)	15 (44.1)	57 (50.4)	12 (44.4)	0.760	0.929	0.790	6 (25.0)	0.164
Good	68 (39.1)	15 (44.1)	42 (37.2)	11 (40.7)				12 (50.0)	
Neither good or bad	20 (11.5)	4 (11.8)	12 (10.6)	4 (14.8)				6 (25.0)	
Bad	-	-	-	-				-	
Very bad	2 (1.1)	-	2 (1.8)	-				-	
**At 12 months post-partum Q3**									
Very good	84 (48.3)	18 (52.9)	53 (46.5)	13 (50.0)	0.819	0.714	0.557		
Good	66 (37.9)	11 (32.4)	46 (40.4)	9 (34.6)					
Neither good or bad	16 (9.2)	4 (11.8)	9 (7.9)	3 (11.5)					
Bad	6 (3.4)	1 (2.9)	5 (4.4)	-					
Very bad	2 (1.1)	-	1 (0.9)	1 (3.8)					

Test for difference between groups at Q3 (Pearson’s chi-square).

^1^‘No pain’ denotes respondents reporting remission of LBP/PGP at Q3.

^2^‘Recurrent pain’ denotes respondents reporting recurrent LBP/PGP at approximately 14 months after pregnancy at Q3.

^3^‘Continuous pain’ denotes respondents reporting continuous LBP/PGP at approximately 14 months after pregnancy at Q3.

^4^‘No pain’ vs. ‘Recurrent pain’, ‘No pain’ vs. ‘Continuous pain’ and ‘Recurrent pain vs. ‘Continuous pain’.

^5^Non-respondents vs. respondents.

*Significance test *p*<0.05.

## Discussion

### Pain status

The overall aim of this study was to identify the prevalence of persistent PLBP and PGP at 12 months postpartum as well as difference over time, evaluating pain status, SRH and current family situation. A significant finding in this present study was an increased probability of ‘recurrent pain’ and ‘continuous pain’ compared to women with ‘no pain’ at Q3 if a woman had experienced LBP before the pregnancy, thus confirming previous findings [20,28]. The most commonly reported pain characters in this study were dull, stabbing and cutting pain, where dull pain seems to be the most common pain character both in women reporting ‘recurrent’ and ‘continuous’ pain at both Q2 and Q3. Previous studies have also shown that these pain characteristics are the most commonly reported among women with PLBP/PGP [3,29]. Women with ‘continuous pain’ at Q3 reported statistically significant higher level of pain compared to women with ‘no pain’ at Q3 at all measured time points, while women with ‘recurrent pain’ (Q3) reported statistically significant higher levels of pain compared to the ‘no pain’ group the past 6 months as well as the past week at Q3. These results were somewhat expected, as it make sense that individuals with recurrent or continuous pain would also report higher levels of pain compared to individuals with no pain at approximately 14 months postpartum. Noteworthy is that pain status appears to change over time but that localisation does not seem to change in the majority of women reporting continuous pain. Few women with continuous pain at Q2 report full remission of symptoms at Q3.

### Self-rated health (SRH)

It is well established that poor SRH is related to pain [30] and reduced SRH may influence LBP [31]. In a prospective study, Svedberg et al. [32] have found that back pain contributed to poor SRH [32]. This study showed that there was a statistically significant difference in regard to SRH between the ‘recurrent pain’ and the ‘continuous pain’ group (categorized at Q3) during pregnancy (Q1) and during the first 6 months postpartum (Q2), where the ‘continuous pain’ group (Q3) seemed to report a less favourably health status. Also, there was an increased likelihood that women with ‘continuous pain’ assessed their health status as ‘poor or quite poor’ compared to women with ‘recurrent pain’ at Q3.

### Relationship satisfaction

Social support has shown to be favourable for both health and welfare, particularly beneficial is marriage satisfaction [33]. For example, research has shown an association between marriage and morbidity and mortality benefits where mortality rates are much higher in unmarried women than for married [33]. In our study, the results show a stable relationship satisfaction throughout the three subgroups, where the majority of individuals rated their relationship satisfaction as ‘good’ or ‘very good’. A study by Albert et al. [28] did not show any difference between groups with or without PGP in regard to marital status. This could possibly be due to the fact that having a baby is usually considered a positive life event and thus have a strengthening effect on the relationship. The vast majority of the women in the study were married or cohabiting. Most women reported a relationship satisfaction of ‘good’ to ‘very good’. There were no differences between the three different subgroups (Q3) at either of measured time points, which indicated a stable relationship satisfaction. Interestingly, a statistically significant difference in relationship satisfaction was shown between respondents and non-respondents, with a less positive scoring regarding relationship satisfaction among the non-respondents in this study.

### General discussion

The findings in this study regarding pain are congruent with research concerning both NSLBP and PLBP/PGP, where recurrence of LBP and PLBP/PGP is strongly correlated with previous episodes of LBP [11-15,20,28]. In addition, previous research in the non-pregnant general population shows that an increase in duration of an episode of LBP and/or persistence is a strong predictor of poor outcome [34,35]. Bothersomeness and psychosocial measures have also been found to be a valid measure of severity in LBP [36] and there appears to be an accumulation of risk over time for pain itself [37].

It has been suggested that PLBP/PGP is to be considered a ‘normal condition’ of pregnancy [24,38] and PGP has been explained by early menarche [39], biomechanical dysfunction in the pelvic joints due to hormonal and postural changes during pregnancy [1,40,41]. Nevertheless, these findings are inconclusive.

Numerous women suffering from PLBP/PGP experience difficulties performing normal daily activities such as prolonged sitting and/or getting up from a sitting position, turning over in bed, dressing/undressing, walking, lifting and carrying small weights [9,42]. Also, women with PGP seem to be more afflicted than women with PLBP [8,16,43] and some may become so incapacitated to the extent that there is a need to use crutches and/or wheelchairs [2,9]. Many women also experience sexual difficulties due to the pain. We have previously reported that 7/10 women with PLBP/PGP are more likely to have an unsatisfying sexual life during pregnancy compared with women without pain [10]. So when taking into account the decreased functional status of many women suffering from PLBP/PGP and that the life-time prevalence of LBP in Swedish women has been estimated to 66% [23], and that the prevalence of PLBP/PGP during pregnancy is even higher (72%) [20], this condition must instead be considered a complication of pregnancy and a major health issue among women in childbearing age.

The results in this study revealed that a spontaneous full recovery with no recurrences of symptoms seems to be an unlikely course for some women suffering from PLBP/PGP, very much like for most non-pregnant individuals [44,45]. Further, this study shows that pain status appears to change over time and for some women the condition is not self-limiting. Instead, 142 out of 176 women (almost 80%) responding to Q3 reported recurrent or continuous pain 14 months postpartum, constituting a prevalence of persistent pain of 22% from the initial cohort (n = 639). These findings can be compared in the light of the research by Norén et al. [18] that observed that 5% of all pregnant women, or 20% of pregnant women with LBP during pregnancy, still experience symptoms three years postpartum [18]. Furthermore, recent research shows that a large proportion of non-pregnant individuals in the general population suffering from LBP, still experiences pain one year after an episode of pain and a majority experiences recurrent pain [11,45]. Hence, LBP can no longer be seen as a self-limiting condition in neither the non-pregnant general population nor in women affected with PLBP/PGP.

A long-standing top priority has been to establish more homogenous subgroups of patients suffering from LBP and several attempts have been made to do so (i.e. subgrouping based on pain severity and psychosocial characters). Lately, several researchers have focused on different trajectories in the natural and clinical course of LBP to enable the identification of clinically meaningful subpopulations [46-49]. The result in this study suggests that women suffering from recurrent or continuous PLBP/PGP may very well constitute a specific prognostic category of patients, even though further research is needed. Additionally, women with recurrent or continuous PLBP/PGP postpartum may also need earlier interventions and more specific treatment regime for better management of their symptoms, as a more conservative pain management approach may be counterproductive in regard to symptomatology.

In the field of LBP research, predictors of poor outcome has shown to be, but not limited to, high pain intensity, long duration, distress, low self-efficacy and previous LBP [50,51]. Low scores regarding SRH may also influence LBP [31]. However, there is one risk factor that has been suggested to be of particular importance and that is previous episodes of LBP [52] and this is also true for PLBP and/or PGP [1,2,53]. This study confirms previous findings by demonstrating that women who have experienced LBP before their pregnancy had an increased likelihood of experiencing recurrent or continuous pain 14 months postpartum. Furthermore, women with ‘continuous pain’ experienced statistically significant higher levels of pain at all measured time points compared to ‘no pain’ and the ‘recurrent pain’ group.

### Methodological considerations

There are some methodological considerations in this study that should be acknowledged. Today, PGP is defined in accordance with positive diagnostic tests as well as pain upon palpation of the ligaments and joints of the pelvis [1] and the pain can be continuous or recurrent. However, this study commenced in 2002 and at that point in time the above definition was not available. Instead pain drawings were used to describe pain location [20]. PGP has often been identified and confirmed by self-rated pain location and/or in combination with clinical tests [6,8,9] and PLBP and PGP can be distinguished from each other through pain locations and clinical examinations [8]. However, lumbar pain symptoms could not be excluded in this study since pain sites correlates with common anatomical location of LBP. Nevertheless, the prevalence of LBP are considered stable, while pelvic pain increases [54] during pregnancy, thus determinants and outcomes are mostly related to pregnancy-related pelvic pain [21].

A five category alternative is commonly used regarding questions concerning SRH to improve the ability to differentiate self-rated health status among people. However, response alternatives seem to differ between studies [30,32,55,56]. Svedberg et al. [32] used the response alternatives ‘excellent’ , ‘good’ , ‘moderate’ , ‘fairly poor’ , and ‘poor’ while the Swedish National Institute of Public Health use the response alternatives ‘very good’ , ‘good’ , ‘fair’ , ‘bad’ , and ‘very bad’. This could be considered a limitation in this study. Nevertheless, studies using similar response alternatives as in this present study found strong correlations between poor SRH and mortality [55,56], which may indicate that the results regarding SRH in this study is reliable.

The validation of the data in this study has previously been discussed at length [20]. Briefly, the non-respondents did not differ from respondents in regard to maternal age, gestational age, birth weight, mode of delivery, total experience of delivery, epidural or spinal anaesthesia during delivery, and pre-pregnancy or end-pregnancy BMI at Q1. The conclusion was that the data collected through Q1 seem to be representative for women with persistent LBP and/or PGP postpartum. Even though this study is a long-term follow-up study based on a previous cohort study, questions in Q2 and Q3 was similar from those in Q1. In addition, there seem to be no difference between the respondent and non-respondents in regard to base line variables (with the exception of smoking and maternal age at first delivery). Therefore, the data seem to be representative for Swedish women with recurrent or continuous LBP and/or PGP 14 months postpartum.

As with all musculoskeletal pain, psychosocial factors appear to exacerbate the clinical component of pain [57,58]. In addition, a study has shown that postpartum depressive symptoms are three times more prevalent in women with lumbopelvic pain compared to those without [59]. However, the material in this study did not contain information in regard to psychosocial factors (such as self-efficacy, distress, depression and fear-avoidance beliefs) apart from relationship satisfaction and family situation, which are in and by itself a limitation.

### Clinical implications

PLBP/PGP constitutes a significant health problem for many women during and after pregnancy. The main findings in this study suggest that PLBP/PGP is not only a major health problem among women 14 months postpartum, negatively affecting their SRH, but also a major public health issue. In general, women reporting ‘continuous pain’ reported poorer SRH compared both to women with ‘recurrent pain’ as well as ‘no pain’. In addition, women with ‘continuous pain’ reported more of a dull pain at both Q2 and Q3 compared to women with ‘recurrent pain’ and most women with ‘continuous pain’ reported no change of the localisation of pain. This may indicate that there is a difference among women who reported PLBP/PGP during pregnancy regarding the long term clinical outcome and that for some of these women the long-term outcome is less favourable. Thus, screening women with risk factors for postpartum PLBP/PGP, such as previous LBP, need to be considered early in the pregnancy. This to enable clinicians to provide better pain management, such as i.e. pelvic belt [60], referral to acupuncture and stabilizing exercises [61], and chiropractic care [17,62] but also to facilitate a more realistic view regarding the prognosis of recurrent and continuous PLBP/PGP postpartum.

## Conclusions

On the basis of the findings in this study, we conclude that previous LBP before pregnancy is a strong predictor for recurrent and continuous pain 14 months postpartum (Q3). High levels of pain during pregnancy and during the first 6 months postpartum (Q2) also indicate a poor outcome for women with PLBP/PGP at 14 months postpartum (Q3). Furthermore, poor SRH was more common among women with ‘continuous pain’ during pregnancy and 6 months postpartum (Q2) compared to women with ‘recurrent pain’. The perceived relationship satisfaction reported in this study appears to be considered ‘very good’ or ‘good’ with a good stability between the groups. Currently, knowledge of the long-term effect and life situation for women with PLBP/PGP is limited. Future studies concerning the long-term effect PLBP/PGP after pregnancy should be conducted to further investigate risk factors for persistent PLBP/PGP postpartum in order to improve intervention outcomes and pain management.

## Competing interests

The authors declare that they have no competing interests.

## Authors’ contributions

CB was involved in analysis and interpretation of the data, drafting and revising of the manuscript and has given final approval. MP was involved in the interpretation of data and revision of the manuscript and gave final approval. IM was involved in study design, data collection, revision of the manuscript, and interpretation of data and gave final approval. All authors read and approved the final manuscript.

## Pre-publication history

The pre-publication history for this paper can be accessed here:

http://www.biomedcentral.com/1471-2393/14/48/prepub
